# *Drosophila* as a Model to Study the Mechanism of Nociception

**DOI:** 10.3389/fphys.2022.854124

**Published:** 2022-03-28

**Authors:** Jianzheng He, Botong Li, Shuzhen Han, Yuan Zhang, Kai Liu, Simeng Yi, Yongqi Liu, Minghui Xiu

**Affiliations:** ^1^Provincial-Level Key Laboratory for Molecular Medicine of Major Diseases and the Prevention and Treatment with Traditional Chinese Medicine Research in Gansu Colleges and University, Gansu University of Chinese Medicine, Lanzhou, China; ^2^College of Basic Medicine, Gansu University of Chinese Medicine, Lanzhou, China; ^3^Key Laboratory for Transfer of Dunhuang Medicine at the Provincial and Ministerial Level, Gansu University of Chinese Medicine, Lanzhou, China; ^4^College of Integrated Traditional Chinese and Western Medicine, Gansu University of Chinese Medicine, Lanzhou, China; ^5^State Key Laboratory of Animal Nutrition, College of Animal Science and Technology, China Agricultural University, Beijing, China; ^6^College of Public Health, Gansu University of Chinese Medicine, Lanzhou, China

**Keywords:** nociception, conserved genetics, nociceptive sensory neurons, behavioral assay, *Drosophila melanogaster*

## Abstract

Nociception refers to the process of encoding and processing noxious stimuli, which allow animals to detect and avoid potentially harmful stimuli. Several types of stimuli can trigger nociceptive sensory transduction, including thermal, noxious chemicals, and harsh mechanical stimulation that depend on the corresponding nociceptors. In view of the high evolutionary conservation of the mechanisms that govern nociception from *Drosophila melanogaster* to mammals, investigation in the fruit fly *Drosophila* help us understand how the sensory nervous system works and what happen in nociception. Here, we present an overview of currently identified conserved genetics of nociception, the nociceptive sensory neurons responsible for detecting noxious stimuli, and various assays for evaluating different nociception. Finally, we cover development of anti-pain drug using fly model. These comparisons illustrate the value of using *Drosophila* as model for uncovering nociception mechanisms, which are essential for identifying new treatment goals and developing novel analgesics that are applicable to human health.

## Introduction

Pain is an “unpleasant sensory and emotional experience associated with actual or potential tissue damage”—as defined by the International Association for the Study of Pain^1^, it is an indispensable and rich sensory experience which can help people promote the healing and improvement of the injured or diseased parts of the body (Young et al., 2012). Pain can be classified into nociception, inflammatory and pathological pain according to the pathological mechanism (Costigan et al., 2009; Woolf, 2010; Haanpaa et al., 2011), and acute, chronic, and occasional pain according to the duration (Turk, 2001; Glare et al., 2019). Pain is a subjective experience, and nociception is an objective neural process that encodes and processes harmful stimuli. It is an evolutionary conservative mechanism that reminds organisms of potential tissue damage and is vital to survival (Neely et al., 2010; Khuong and Neely, 2013). For living organisms, the rapid response to harmful stimuli and the ability to react and avoid them (pain/nociception) is crucial (Milinkeviciute et al., 2012).

Most of the research objects on nociception are model animals, such as monkey (Lee et al., 2007), mice (Luo et al., 2019), zebrafish (Malafoglia et al., 2013), *Drosophila melanogaster* (Leung et al., 2013), *C. elegans* (Nkambeu et al., 2020) and so on. As a bridge between disease research and human beings, model animals play an important role in modern medical research. Extensive modeling has been performed in mammals, however, these models are expensive, and have ethical implications. In contrast, *Drosophila* has its unique advantages as a model animal for pain research, small size and relatively short lifecycle allows it could be produced in large numbers and easy to work with. Most importantly, fruit flies show a high degree of homology with humans at the organ and gene level, with flies sharing functional counterparts for most organ systems (Fortini et al., 2000; Chien et al., 2002). It has been estimated that 75% of human disease genes have conserved homologs in *Drosophila*, making this fly a model organism of great potential (Bier, 2005). Using fruit flies as human disease model would avoid the ethical controversy. At present flies has been used extensively as a model for human disease already, for example, to study cancer, Alzheimer’s disease, nociception, obesity and diabetes and so on (Milinkeviciute et al., 2012; Enomoto et al., 2018; Tsuda and Lim, 2018; Warr et al., 2018).

As previously mentioned, there are conserved physiological mechanisms underlying the nociceptive system between human and flies (Sneddon, 2018). The nociceptors in the primary afferent nerve fibers are stimulated by thermal, mechanical and chemical stimulation, converted into electrical signals, and then transmitted to the central nervous system such as the spinal cord, and finally felt the pain (Julius, 2013; Bourne et al., 2014; Dai, 2016; Sneddon, 2018; St, 2018). These nerve fibers quickly transmit the perceived harmful information to the central nervous system through action potentials. In this process, ion channels play a vital role. These ion channels are specifically expressed in the above-mentioned nerve fibers (Julius, 2013; Dai, 2016; Yam et al., 2018). TRP, Piezo and other ion channels have been identified as key pain receptors (Hwang and Oh, 2007; Volkers et al., 2015). Among these channels, TRPV1, TRPA1, Piezo1 and Piezo2 are expressed in nociceptors (Liedtke, 2007; Flood et al., 2013; Volkers et al., 2015; Himmel and Cox, 2017; Boonen et al., 2021). They serve as detectors and sensors for cold, heat, chemical and mechanical stimuli in nociceptors. These conserved genes in *Drosophila* well prove the potential of flies as a nociceptive model animal. The purpose of this review is to present the aggregate findings of the pain-related genes in order to discuss the possibilities for *Drosophila* as model animal in nociception research, and provide a comprehensive evaluation for future human nociception studies.

## The Role of Pain-Related Genes in Regulating Nociception/Pain

### Transient Receptor Potential Channels

Transient receptor potential (TRP) channels are a large family of ion channels, and most of them are conserved from *Drosophila* to humans. It has more than 50 subtypes, divided into 7 subfamilies according to their amino acid sequence homology, which includes vanilloid (TRPV1-6), canonical or classic (TRPC1-7), melastatin (TRPM1-8), non-mechanoreceptor potential C (NOMP-like, TRPN1), long TRP ankyrin (TRPA1), polycystins (TRPP1-5) and mucolipins (TRPML1-3) (Clapham et al., 2001; Nilius et al., 2005, 2012; Wu et al., 2010; Li, 2017; Bamps et al., 2021). TRP channels allow an inward cation current to regulate cell function, and have a variety of activation modes by mechanical, thermal and chemical stimuli (Nilius et al., 2007; Nilius and Owsianik, 2011). Therefore, the TRP family has a variety of physiological functions, including vision, hearing, taste, thermosensation and response to different environmental stimuli (Gees et al., 2012). The TRP channels are probably best known for its role in nociceptive perception, it is the largest group of noxious ion channels involved in pain. Among the sub-families, TRPA1, TRPV1, and TRPM2 have been shown to be related to nociception (Table 1; Flood et al., 2013; Himmel and Cox, 2017; Maiese, 2017; Logashina et al., 2019).

**TABLE 1:** Genes that regulate pain in humans and *Drosophila*.

Human genes	Regulated types of pain	References	Drosophila genes	Regulated types of pain	References
TRPA1	Neuropathic pain, nociception, allodynia, cold hyperalgesia	Yu et al., 2010; Fowler and Montell, 2013; Hehlert et al., 2021	dTRPA1	Thermal nociception	Kolisek et al., 2005; Zhong et al., 2010; Khuong and Neely, 2013; Glare et al., 2019
			Painless	Thermal and mechanical nociception	Lee et al., 2005; Kwan and Corey, 2009; Zhong et al., 2010
			Pyrexia	Thermal nociception	Liedtke, 2007; Zhong et al., 2010
TRPM2	Thermosensation and nociception inflammatory, neuropathic and chronic pain	Hwang and Oh, 2007; Honjo et al., 2012; Zygmunt and Hogestatt, 2014			
Piezo1	Promotes mechanical response	Ma and Quirion, 2007; Maiese, 2017	DmPiezo	Mechanical nociception	McClung and Hirsh, 1998; Mauthner et al., 2014; Luo et al., 2019; Massingham et al., 2021
Piezo2	Feel gentle touch, proprioception, and abnormal tactile pain	Manev et al., 2003; Manev and Dimitrijevic, 2004; Malafoglia et al., 2013; Mandel et al., 2018	piezo-like	Crawling pattern and body gesture control	McParland et al., 2021
ASIC3	pain caused by acid	Merritt and Whitington, 1995; Milinkeviciute et al., 2012; Luo et al., 2017	Pickpocket1	Mechanical nociception	Minke et al., 1975; Neely et al., 2010; Nassini et al., 2014; Murthy et al., 2018
			Pickpocket26	Mechanical nociception	Neely et al., 2010, 2011
			Pickpocket30	Mechanical nociception	Nichols et al., 2002

TRPA1 is a channel with non-selective permeability to calcium, sodium and potassium, while its permeability to calcium is higher than that of other TRPs (Kwan and Corey, 2009). It acts as a sensor for cell damage signals and is involved in inflammation and immune response (Bandell et al., 2004; Kwan et al., 2006; Kwan and Corey, 2009; Neely et al., 2011; Zygmunt and Hogestatt, 2014; Laursen et al., 2015; Viana, 2016). Mostly, TRPA1 is crucial in mediating long-term hypersensitivity to thermal, cold, chemical, and mechanical stimuli detected in nociceptive, inflammatory, and neuropathic pain models (Story et al., 2003; Corey et al., 2004; Kwan et al., 2006; Karashima et al., 2009; Del et al., 2010). TRPA1 has been proposed to function as a temperature-insensitive detrimental heat sensor and a detrimental cold sensor (Laursen et al., 2015). TRPA1 promotes excitatory effects of bradykinin through the PLC/calcium signaling pathway (Bandell et al., 2004), so it is an important downstream target for inducing pain receptor hypersensitivity (Bautista et al., 2006). In inflammatory pain, the role of TRPA1 channels is two sides. On the one hand, pro-inflammatory factors activate nociceptors through TRPA1. On the other hand, TRPA1 stimulation is usually related to the release of pro-inflammatory neuropeptides (Nassini et al., 2014).

TRPV1 is a cation permeable channel and shows important influence in feeling nociceptive stimuli and producing pain in primary afferent nociceptors (Immke and Gavva, 2006). As an ion channel, it can be activated by specific activators, such as vanillin, capsaicin, sorbamide, etc., (Gees et al., 2012; Julius, 2013; Dai, 2016; Li, 2017; Hung and Tan, 2018). TRPV1 is now considered to be a molecular integration factor of pain stimuli, and drug target. In animals, the sensitization and activation of peripheral nociceptors can cause TRPV1 to transmit nociceptive signals to the central nervous system, thereby producing unpleasant and painful sensations, warning the body of potential harmful threats (Hung and Tan, 2018). TRPV1 not only plays a vital role in nociception, but also leads to the generation of action potentials during inflammation, which in turn leads to the generation of pathological pain, such as thermal hyperalgesia, spontaneous pain and mechanical hypersensitivity (Caterina et al., 2000; Ma and Quirion, 2007). TRPV1 knockout mice have a significant reduction in thermal hypersensitivity after tissue injury, which clearly proves that TRPV1 is involved in the development of inflammatory pain (Caterina et al., 2000; Davis et al., 2000). TRPM2 as a calcium ion-permeable non-selective cation channel is expressed in the peripheral nervous system and immune system, which is activated by oxidative stress, moderate temperature and intracellular adenosine diphosphate ribose (ADPR) in various types of cells (Kolisek et al., 2005; Togashi et al., 2006). TRPM2 is of great importance in the pathogenesis of inflammation and neuropathic pain (Eisfeld and Luckhoff, 2007; Faouzi and Penner, 2014). A study showed that in carrageenan-induced inflammatory pain and sciatic nerve injury-induced neuropathic pain models, TRPM2 knockout mice have alleviated mechanical hyperalgesia and thermal hyperalgesia (Di Meglio et al., 2004).

The first evidence for the existence of TRP channels were found in *Drosophila* flies. Cosens and Manning used electroretinogram (ERG) measurements to analyze a spontaneous mutant in *Drosophila* that exhibited a temporary rather than a continuous response under long-term bright light (Cosens and Manning, 1969). It is firstly named a “type A” mutation. Later, it was found that this mutant had defects in light transmission, and had a representative name: “transient receptor potential” or Trp (Minke et al., 1975). TRP channels are diverse in structure and can modulate transduction of thermal, mechanical, and chemical stimuli and also can regulate cell growth, cell differentiation, and vascular physiology in flies (Pazienza et al., 2014; Maiese, 2017). The *Drosophila* genome contains genes encoding 13 TRP channels, and encodes four TRPA homologs: *dTRPA1*, *painless*, *pyrexia*, and *water witch* (Fowler and Montell, 2013). TRPA channels have been the most widely studied for their roles in temperature-sensing behavior in flies (Goodman, 2003; Xu et al., 2006; Neely et al., 2011; Bellemer, 2015) and also play important roles in chemical and mechanical sensing (Mandel et al., 2018; Boonen et al., 2021). TRPA1 has been implicated as a mammal noxious cold receptor, which is activated by extremely cold temperatures (below < 15°C) (Kwan and Corey, 2009). However, the TRPA1 homologs *dTRPA1*, *painless*, *pyrexia* in flies have no function in regulation of cold avoidance. The temperature-sensitive diversity of TRPA1 channels in flies and mammals makes researchers more cautious when dissecting the role of TRPA1 in thermal stimulation and screening anti-pain drug using *D. melanogaster*.

dTRPA1 was first identified as a heat-activated channel in flies (Rosenzweig et al., 2005; Kwon et al., 2008). It is 32% identical and 54% similar to its mammalian orthology by amino acid identity, and is activated in response to high temperature, reactive chemicals and downstream of intracellular signaling pathways (Neely et al., 2011; Bellemer, 2015; Boonen et al., 2021). The dTRPA1 channel is expressed in the multiple groups of central neurons and several classes of peripheral sensory neurons (Hamada et al., 2008; Kang et al., 2010; Kim et al., 2010). dTRPA1 participates not only in the thermal pain of adults, but also in the thermal pain of larvae (Neely et al., 2011; Luo et al., 2017). Control adults flies respond very quickly to harmful heat at 46°C, but dTRPA1 mutants respond slowly to harmful heat, and their thermal pain ability was significantly reduced (Neely et al., 2011). Fly larvae trigger noxious rolling behavior when the temperature is below 40°C, and the frequency of this behavior increases rapidly as the temperature rises until 33°C. The above-mentioned nociceptive behaviors all depend on the dTRPA1 channel, the activity of which responds to the rate of temperature change (Luo et al., 2017). In addition to participating in the response to harmful temperature stimuli, dTRPA1 has also been detected in chemical stimuli (Tracey et al., 2003; Im and Galko, 2012). Boonen et al. (2021) found that wild-type flies avoid citronellal and menthol in olfactory tests, while dTRPA1 mutant flies have reduced this behavior. dTRPA1 channel mediates chemical avoidance in gustatory receptor neurons (GRNs), in which know down of *dTRPA1* in GRNs significantly reduced the aversive response to aristolochic acid (Kim et al., 2010).

*Painless* as a member of the TRPA family channel was discovered and identified as an important gene for thermal and mechanical nociception in *Drosophila* flies (Tracey et al., 2003; Xu et al., 2006). It is expressed in the larval peripheral nervous system (Tracey et al., 2003), and various regions of the adult brain, such as mushroom body, a region important for learning and memory (Busto et al., 2010) elliposoid body of the central complex (Sakai et al., 2012, 2014); olfactory projection neurons in antennal lobes (Wang et al., 2011); and the pars intercerebralis including insulin-producing cells (Sakai et al., 2012, 2014). Painless is a molecular sensor for noxious thermal stimuli in larvae and adult flies (Goodman, 2003). Studies have confirmed that wild-type larvae exhibit typical “rolling behavior” within 1 s of being contacted by the heated probe above 40°C, and this activity is absent in the *painless* mutant (Goodman, 2003). When the heating temperature exceeds 38°C, the firing of the multidendritic sensory neurons increases, but this increase is not seen in the *painless* mutant (Xu et al., 2006; Sokabe and Tominaga, 2009). In the hot plate assay, *painless* mutant adults exhibited a behavioral defect and could not jump quickly to escape from a hot plate, which can be rescued by a transformed *painless* gene, indicating that painless is required for thermal nociception in adult flies (Xu et al., 2006). Painless requires Ca^2+^ as a co-agonist for heat-evoked activation. Painless failed to respond to heat in the absence of intracellular and extracellular Ca^2+^ (Liu et al., 2003). Painless is also required for chemical and mechanical nociception (Tracey et al., 2003; Al-Anzi et al., 2006; Mandel et al., 2018). Mandel et al. (2018) found that allyl isothiocyanate (AITC) remarkably reduce the proboscis extension reflex frequencies in wild-type genotypes but did not in *painless* mutant, and AITC evoked calcium changes in *painless* expressing neurons. Expression of painless was also detected in mechanically-sensitive Johnston’s organ, while painless could be activated by mechanical stimuli (Tracey et al., 2003). Additionally, painless is involved in a variety of neural processes in flies including negative geotaxis (Sun et al., 2009), larval social behavior (Xu et al., 2008) and sexual receptivity of virgin females (Sakai et al., 2014).

*Pyrexia* (*pyx*) gene is a heat-sensitive TRPA channel and protects flies from high temperature stress (Lee et al., 2005; Xu et al., 2006; Hamada et al., 2008). It is ubiquitously expressed along the dendrites of a subset of peripheral nervous system neurons and is more permeable to K^+^ than to Na^+^ (Lee et al., 2005). 60% of pyx null flies were paralyzed within 3 min after exposure to 40°C, while applying the same stimulation to wild-type fruit flies, the number of paralysis was only 9% (Lee et al., 2005). *pyx* is also responsible for the response of temperature-sensitive anterior cell (AC) brain neurons, which regulate the temperature preference behavior of adult flies (Hamada et al., 2008; Tang et al., 2013). *pyx* is involved in temperature synchronization of circadian clocks, in which pyx mutants fail to synchronize their behavior to temperature cycles between “night” and “day” (Wolfgang et al., 2013).

### Piezo Channel

Mechanical transduction is the process of converting mechanical force into biological signals, which plays a key role in various physiological processes of animals (Volkers et al., 2015). It is through the mechanically sensitive cation channel converts the mechanical stimulus received by the animal body into various activities, and plays an important role in the regulation of touch, hearing and blood pressure (Bagriantsev et al., 2014). Piezo channel is a type of mechanical-sensitive ion channel, which is necessary for cells to respond to mechanical stimuli (Coste et al., 2010). In vertebrates, Piezo channel proteins mainly include Piezo1 and Piezo2 proteins, which are encoded by the genes *Piezo1/FAM38A* and *Piezo2/FAM38B*, respectively (Coste et al., 2010, 2012; Bagriantsev et al., 2014). Piezo1 channels are characterized by slower kinetics, and can react to more persistent activation (Lewis et al., 2017). After silencing Piezo1 expression in chondrocytes, the number of chondrocytes responding to mechanical stimulation decreased, while activating Piezo1 significantly promotes mechanical response in chondrocytes (Servin-Vences et al., 2017). Piezo2 as a faster kinetics are more specified for detection of transient mechanical forces (Ranade et al., 2014; Woo et al., 2015; Szczot et al., 2018). Recent studies have shown that Piezo2 is essential for mediating abnormal tactile pain in mice (Murthy et al., 2018; Szczot et al., 2018), and it has been confirmed that Piezo2 is also necessary for humans to feel gentle touch, proprioception, and abnormal tactile pain (Szczot et al., 2018).

Only one single Piezo protein was found in lower organisms, such as nematodes and fruit flies (Hamada et al., 2008). In the *Drosophila melanogaster*, there is only one copy of the force-gated ion channel, Dm*Piezo*, a Ca^2+^ permeable non-selective cation channel, similar to its mammalian homolog (Coste et al., 2012). Dm*Piezo* is 24% identical to mammalian piezos, with sequence conservation throughout the length of the proteins (Coste et al., 2012). Studies have shown that the expression of DmPiezo is detected in all types of sensory neurons and some non-neural tissues of flies, including multimodal nociceptors of larvae. Among these neurons, Dmpiezo has a special contribution to mechanical pain (Kim et al., 2012). The researchers found that Dmpiezo expression in human cells induces mechanically activated currents, similar to its mammalian counterpart (Coste et al., 2012). In Dmpiezo knockout larvae, the behavioral response to harmful mechanical stimuli is severely reduced, while the response to another harmful stimulus or touch is not affected. Knockdown of Dmpiezo in sensory neurons that mediate nociception is sufficient to weaken the response to harmful mechanical stimuli. Dmpiezo and Pickpocket (ppk) are involved in the parallel pathways of ppk-positive cells, while their absence results in mechanical nociception elimination (Kim et al., 2012). Loss of DmPiezo renders class IV sensory neurons unresponsive to harsh touch (Kim et al., 2012) and makes mechanosensitive visceral neurons, which sit in the fly’s brain and innervate the gut, mechanoinsensitive (Wang et al., 2020). DmPiezo also regulate axon regeneration in flies (Song et al., 2019), in which DmPiezo activation during axon regeneration induces local Ca^2+^ transients at the growth cone, leading to activation of nitric oxide synthase and the downstream cGMP kinase foraging or PKG to restrict axon regrowth, while loss of DmPiezo increases axon regeneration of sensory neurons. A second *Drosophila* piezo family member, piezo-like (pzl; CG45783) shares similarity with that of Dm*piez*o and its mammalian homologs Piezo1 and Piezo2 (Hu et al., 2019). Pzl gene expressed in larval chordotonal neurons is required for locomotion of *Drosophila* larvae. The pzl mutant showed severe defects in crawling pattern and body gesture control, which could be rescued by expressing human or mouse Piezo1, suggesting a conserved role the Piezo-family proteins in locomotion (Hu et al., 2019).

### DEG/ENaC Family Channels

Acid-sensitive ion channels (ASICs) are a group of proton-gated ion channels that belong to the degenerin/epithelial sodium channel (DED/ENaC) family. The channel can be activated when the extracellular pH drops below 7.0, or with aprotic ligands at physiological pH levels. The activation of ASICs mainly triggers Na^+^ influx (Waldmann et al., 1997; Yu et al., 2010). ASIC was found to be a major player in human pain caused by acid (Luo et al., 2017). Increasing evidence further indicates that ASIC3 is a molecular determinant of pain-related tissue acidosis in rodent models. Members of the DEG/ENaC family also play a role in nociception, and have been shown to be essential mechanical transduction molecules in *Drosophila* flies (Luo et al., 2019). *Pickpocket1* (*Ppk1*) encodes an ion channel subunit of the DEG/ENaC family and is responsible for mechanical nociception responses in flies (Adams et al., 1998; Zhong et al., 2010). It is widely expressed in nociceptive and class IV multidendritic neurons (Zhong et al., 2010). Another ion channel subunits *balboa* (also known as *ppk26*) is highly enriched in nociceptive neurons and could bind to PPK to regulate mechanical nociception behaviors in *Drosophila* larvae (Guo et al., 2014; Mauthner et al., 2014). *Ppk26* mutant showed severe behavioral defects in a mechanical nociception behavioral test but responded to noxious heat stimuli compared to wild-type larvae (Guo et al., 2014). *Ppk1* and *ppk26* have the same signaling pathway to regulate mechanical nociception, and they do not have functional response to in thermal stimulus (Gorczyca et al., 2014). Ppk30 as a member of the *Drosophila* Ppk family is detected by class IV multidendritic neurons, and has a role in mechanosensation, but not in thermosensation (Jang et al., 2019).

## *Drosophila* Models of Nociception/Pain

*Drosophila* fly has high homology with human disease genes (75%), reproduces rapidly on its own, and the cost of establishing and maintaining a sufficient number of drosophila is much lower than that of the same number of vertebral model animals. These advantages make fly become a tool for studying the conservative genetics of pain (Tracey et al., 2003; Bier, 2005). The fly nociceptors are similar to vertebrates in morphology and function, and they have unique naked nerve endings. The end of the nerve dendrites of *Drosophila* cover the entire epidermis without overlapping, allowing them to quickly perceive tissue damage. This characteristic proves the potential of *Drosophila* as a model animal for noxious research (Grueber et al., 2003, 2007; Hwang and Oh, 2007).

### Nociceptive Sensory Neurons in Flies

Nociception refers to the sensation of harmful stimuli that can cause tissue damage. Nociceptive sensory neurons in *Drosophila* have one axon and one or several dendrites each (Hehlert et al., 2021). Fly larvae have two main peripheral sensory neurons located below the barrier epidermis: type I and type II, according to dendrite number and anatomy (Kernan, 2007). The type I neurons are related to bristle type and chordotonal sensory organs and have a single ciliated dendrites (Hwang et al., 2007). The type I neurons are more involved in mechanical sensory functions, such as light touch (Kernan et al., 1994). The type II neurons have many dendritic extensions that project to nearly every epidermal cell of the larval barrier epidermis, thus the type II neurons are also called multidendritic (md) sensory neurons or dendritic arborization (DA) (Grueber et al., 2002). They are structurally similar to mammalian nociceptors (Gao et al., 1999; Grueber et al., 2002). Larvae with gene-silenced md neurons are completely insensitive to harmful stimuli and cannot produce noxious responses. This underlying evidence suggests that md sensory neurons function as nociceptors (Williams and Truman, 2005; Grueber et al., 2007). The TRP channel mentioned above is necessary for nociception, and it has been confirmed that it is expressed in md neurons, which further shows that the status of md neurons in nociception is crucial (Tracey et al., 2003; Rosenzweig et al., 2005). Morphological studies on type II neurons show that these neurons are not a unified cell population. On the contrary, at least four subtypes have been identified (Grueber et al., 2002).

According to the complexity of dendrites and other morphological characteristics, these neurons are named class I-IV neurons that tile the larval body wall (Merritt and Whitington, 1995; Schrader and Merritt, 2000; Grueber et al., 2007). The dendrites of class I neurons are the simplest, while class IV neurons are the most complex (Grueber et al., 2002). Class I and Class II dendritic domains are relatively sparse and compact, while Class III and Class IV neurons have more complex branching patterns, wider coverage, and no branch overlap (Grueber et al., 2002, 2003). Class I neurons project to the motor nerve stacks of the ventral dorsal ganglia and are thought to provide feedback to the motor neurons. However, class II, class III, and class IV neurons all project to the ventral nerve pile, and by analogy with other insects, they are predicted to have somatosensory functions (Hwang et al., 2007; Yoshino et al., 2017; Burgos et al., 2018). Class I neurons are important for coordinating the appropriate timing of peristaltic locomotion (Cheng et al., 2010). Class II and Class III are both related to light contact reactions, of which type III takes the leading role (Tsubouchi et al., 2012; Yan et al., 2013). Class III neurons also mediate the mechanical nociception and cold nociception (Tsubouchi et al., 2012; Yan et al., 2013; Turner et al., 2016). Class IV neurons appear like mammalian nociceptors morphologically (Tracey et al., 2003) and have polymodal sensitivity to a variety of sensory stimuli (Ohyama et al., 2013). Ablation or silencing class IV neurons significantly eliminates larval responses to noxious stimuli, while activation of class IV neurons is sufficient to stimulate corkscrew-like rolling behavior that is similar as larvae receive noxious stimuli (Tracey et al., 2003; Hwang et al., 2012; Ohyama et al., 2013). These neurons in the peripheral nervous system are responsible for perception of multiple nociceptive modalities, including mechanical force, harmful heat, low-wavelength light, and chemical stimuli, through distinct receptors (Tracey et al., 2003; Kang et al., 2010; Xiang et al., 2010; Hwang et al., 2012; Ohyama et al., 2013). Diverse ion channel are expressed in class IV neurons to evoke depolarization in response to corresponding noxious stimuli (Tracey et al., 2003; Lee et al., 2007; Zhong et al., 2010; Hwang et al., 2012; Kim et al., 2012).

Much of the current research on pain in *Drosophila* flies has focused on nociception, which is similar to acute pain in mammals. When flies suffer from noxious stimuli, multiple pathways are activated in md neurons. This includes the dTRPA1 (Mandel et al., 2018), painless (Tracey et al., 2003) and Pyrexia (Lee et al., 2005) that sense thermal pain; the DmPiezo (Song et al., 2019), painless (Tracey et al., 2003) and Pickpocket families (Zhong et al., 2010) that sense mechanical pain. After acute pain perception occurs, it is often accompanied by prolonged allodynia and hyperalgesia. Multiple pathways related to allodynia and hyperalgesia are also found in md neurons. Hedgehog (Hh) signaling is involved in allodynia and hyperalgesia when *Drosophila* larvae are exposed to UV light (Babcock et al., 2011). Meanwhile, Hh signaling acts in parallel with tumor necrosis factor (TNF) signaling to mediate allodynia (Babcock et al., 2009), and several TRP channels described above mediate allodynia and hyperalgesia downstream of these pathways. Painless is required for the development of Hh- or TNF-induced thermal hyperalgesia, whereas dTRPA1 is required for Hh-induced thermal hyperalgesia (Babcock et al., 2011). The BMP pathway is also expressed in md neurons during allodynia and hyperalgesia, and it is located downstream of the Hh signaling pathway (Honjo and Tracey, 2018). Decapentaplegic (Dpp, mammalian bone morphogenetic protein 2/4 ortholog) and its downstream signaling pathways in *Drosophila* md neurons have also been shown to be required to induce allodynia (Gjelsvik et al., 2018). The above studies show that when pain occurs, related pain signaling pathways in *Drosophila* md neurons are co-expressed to participate in acute nociception and subsequent chronic pain (allodynia and hyperalgesia).

### Different Stimulation of Acute Nociception

Currently, the method of nociceptive research using *Drosophila* fly as a model animal focuses on thermal, cold, chemical and mechanical stimulation of acute nociception.

**FIGURE 1:**
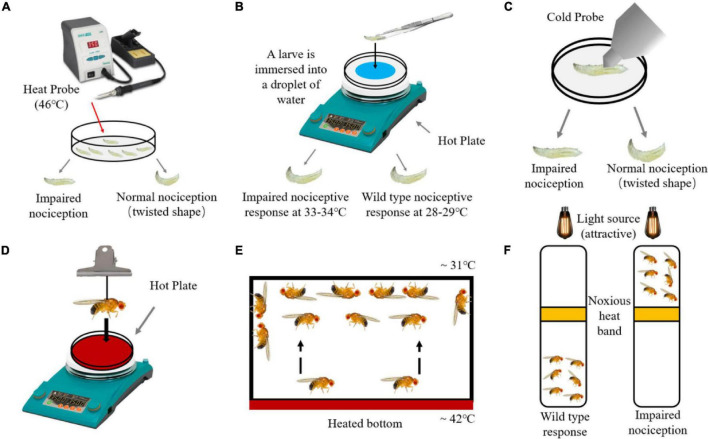
Detection method of harmful temperature nociception in *Drosophila*. **(A)** When using a 46°C heat probe to contact *Drosophila* larvae, the rolling reaction time of wild-type larvae earlier than the larvae with impaired nociception. **(B)** *Drosophila* larvae with impaired nociception and wild-type larvae can cause rolling reaction when placed in water at 33–34 and 28–29°C respectively. **(C)** When using a cold probe to contact larvae, the rolling reaction time of wild-type larvae earlier than the larvae with impaired nociception. **(D)** After keeping the adult flies on a hot plate at 47°C, record the incubation period of the *Drosophila* to produce a jumping response. **(E)** Place the adult flies in a heating device, and wild-type flies will concentrate on the upper part of the device with a suitable temperature. **(F)** The optical drive heat avoidance test uses a heated aluminum ring as a harmful barrier between the adult *Drosophila* and the light source (attractive).

Regarding the experimental example of thermal nociception, fly larvae and adults have different methods. One of the most classic experimental examples is to collect fly larvae and place them in a petri dish, touch it with a soldering iron heated to 46°C, then the wild-type larvae would make a rolling response in a very short time (Tracey et al., 2003; Petersen et al., 2018; Figure 1A). TRPA1 mutants or painless mutants will exhibit a markedly slow response to temperature. The above experimental model has opened the door to the study of nociception or pain in *Drosophila* (Tracey et al., 2003; Manev and Dimitrijevic, 2004; Babcock et al., 2009; Aldrich et al., 2010; Coste et al., 2010). Another way to study the heat damage of larvae is to pour water on a petri dish filled with agar to form a water film so that the larvae can roll freely, put the larvae in the petri dish, and then put the petri dish on the heating plate. Record the length of time for the rolling response of larvae at different temperatures (Oswald et al., 2011; Figure 1B). Flies cannot be exposed to low temperatures for a long time, and their behavior prefers warmer temperatures, but the mechanism by which they perceive and avoid cold stimuli has not been studied until recently. The method of measuring the cold nociception of *Drosophila* larvae is to place the cold probe at a 45° angle to the back of the larva, and apply enough pressure downward to make the surface of the larva slightly concave while allowing it to move forward or backward (Turner et al., 2017). Keep the cold probe still for 10 s until the cold-induced response of the Drosophila larvae is observed, which is the response latency period (Figure 1C). The response latency period is simply the reaction time to cold stimuli. The shorter the latency period, the more sensitive the flies to cold stimuli. The response latency period of wild-type flies is shorter than that of mutant flies’ in the revised manuscript.

The detection of typhoid fever on adult *Drosophila* is time-consuming and laborious. Firstly, researchers developed a method to model the “jumping” reflections that flies exhibit when exposed to noxious heat (Figure 1D), in which flies is suspended on an electric heating plate (47°C) using a nylon rope, then is dropped to get in touch with heating plate, and waiting time for flies to jump is recorded (Xu et al., 2006). Secondly, the adult flies are placed in an incubator with a heating function at the bottom, and the bottom of the box is heated to 46°C (Figure 1E). Wild-type flies will avoid that surface and rest at the upper part of 31°C (Neely et al., 2010). The group should be used as the nociception detection unit, and it is required to be simple and effective. Researchers can use this device to identify genes related to thermal nociception. The third method is to combine the light preference response of adult flies with harmful heat avoidance (Figure 1F). Flies are placed in a vertical transparent device, and a heating aluminum ring and a lamp are placed on the middle and top of the device. Wild-type flies with normal receptors are not attracted by light, while flies with knockdown of *painless* are attracted by light and pass through the heated aluminum ring (Benzer, 1967; Aldrich et al., 2010).

**FIGURE 2:**
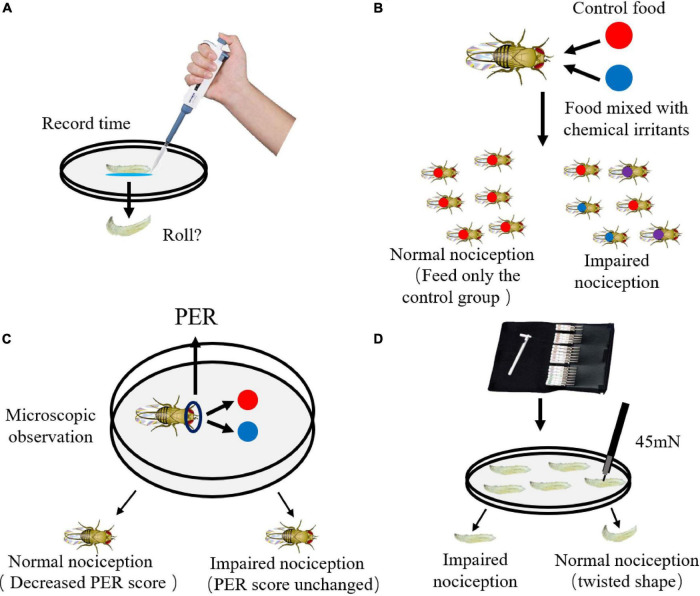
Detection method of chemical and mechanical nociception in *Drosophila*. **(A)** Use a pipette to place the chemical stimulus around the body of the larvae and record the incubation period. **(B)** Adult flies with impaired nociception eats indiscriminately, while wild-type flies eat only control food. **(C)** Provide two kinds of food and record the PER score of adult flies. **(D)** The von Frey fiber was calibrated and used for the determination of mechanical nociception: wild-type adult flies will have a rolling response when the force exceeds 45 mN, and the adult flies with impaired nociception have a greater rolling force.

The way to study the chemical stimulation of fruit flies is to add nociceptor activators, such as capsaicin, menthol, allicin, isothiocyanate, etc., to food, which cause flies to resist food (Al-Anzi et al., 2006; Kim et al., 2010; Li et al., 2020; Figure 2A). Briefly, third-instar larvae are placed in a petri dish, use a pipette to add the above-mentioned chemical stimulus solution under and around the flies, and record the incubation period of the fruit flies (the time between the addition and the tumbling behavior) (Lopez-Bellido et al., 2019). As the concentration of the solution increases, the incubation period will become shorter and shorter. Another way to determine chemical stimulation is to test food choice, flies can make choice between control food and food with chemical irritants at the same time, and the chemical irritants can be increased in dose (Figure 2B). The control food is marked with red dye, and the food with chemical stimulus is marked with blue dye. The abdomen of wild-type flies will show a single red color, while the abdomen of mutant flies will show three colors, red, blue, and purple (two groups of food eat at the same time) (Al-Anzi et al., 2006). A method similar to the above method is to use the *Drosophila*’s proboscis extension response (PER) as an indicator of whether flies eat (Figure 2C). PER is judged based on the reaction of the nose of hungry flies when they eat normal food. Adding chemical stimulants to food will reduce the PER score of wild-type flies (Al-Anzi et al., 2006; Kang et al., 2010).

The noxious rolling response of fruit flies to harmful mechanical damage is produced by stimulating von Frey fibers in a petri dish (Figure 2D; Tracey et al., 2003). The mechanical stimulation is provided by the calibrated von Frey fiber, the larvae are less active, and the noxious response is easy to evaluate, so this method is not easy to be applied to the adult mechanical damage study. First, pour clean water into a petri dish with agar so that the animals can crawl and perform rolling behaviors freely. The larvae will pause their normal feeding behavior when touched. Normal larvae elicit a rigid rolling response when subjected to a force of 45 mN von Frey fibers (Tracey et al., 2003; Hoyer et al., 2018), and painless mutant larvae appeared only spiral coiling until the stimulation increased to 100 mN (Tracey et al., 2003). This method has been improved recently. The von Frey fiber is replaced with a custom-made metal Nitinol (Nitinol) wire probe that detects mechanical damage (Hoyer et al., 2018).

Optogenetics is a powerful tool that enables spatiotemporal control of neuronal activity and circuits in behaving animals. Optogenetic nociception assay is widely used in *Drosophila* fly larvae (Hwang et al., 2007; Honjo et al., 2012; Dannhauser et al., 2020). The optogenetic technique with ChR2::YFP is developed and used to demonstrate the md neurons are nociceptive sensory neurons whose activation is sufficient to trigger larval nocifensive escape locomotion (Hwang et al., 2007; Honjo et al., 2012). Briefly, virgin female flies of the GAL4 driver strain that target md neurons are crossed to male flies of the UAS-ChR2::YFP strain. The larval progeny are allowed to develop and feed on the yeast paste (either atr+ or atr-) for 4 days. For behavioral analysis, the larvae are transferred to plastic Petri dishes and then stimulated with blue light (460–500 nm). Blue light pulses are manually controlled and lasted for several seconds. Nocifensive roll and nocifensive escape locomotion are videotaped and analyzed. This model can be used to dissect the molecular mechanisms that sensitize responses of nociceptors and nociception behaviors (Honjo and Tracey, 2018).

### Chronic Pain Perception in Flies

The above mentioned methods are mainly used to study acute nociception. Acute nociception is often caused by noxious stimuli, which usually protect the animal body from possible harm (Bell, 2018). Chronic pain results from maladaptive changes to this nociceptive system and persists even after the healing process is complete (Voscopoulos and Lema, 2010). Much of what is currently involved in the study of chronic pain in *Drosophila* flies is caused by nerve damage and inflammation following noxious stimuli, which can lead to hyperalgesia (increased sensitivity to noxious stimuli) and allodynia (perceives innocuous stimuli as noxious) (Hamoudi et al., 2018; Khuong et al., 2019).

**FIGURE 3:**
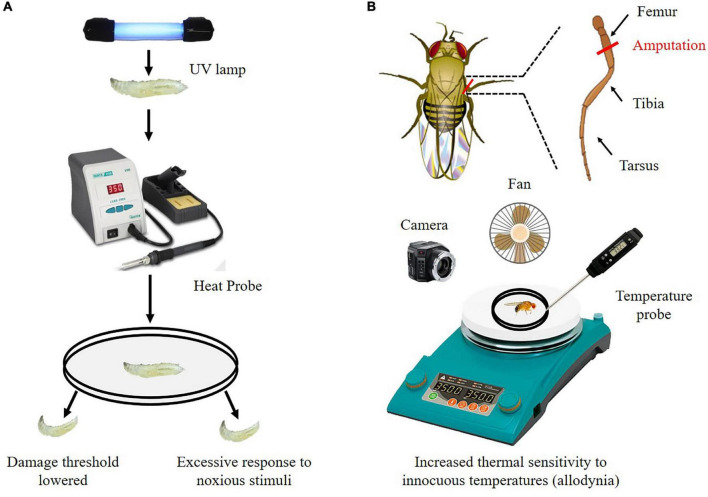
Detection method of chronic pain perception in flies. **(A)** *Drosophila* larvae have a reduced nociceptive temperature threshold and overreact to noxious temperature stimuli after exposure to UV light. **(B)** After amputation, adult flies were subjected to thermal stimulation. Adult flies have increased thermal sensitivity to innocuous temperatures (allodynia).

The chronic pain perception has been explored in larvae for several years. The researchers used ultraviolet (UV) light to induce tissue damage in fruit fly larvae, and then used thermal probes to demonstrate that the tissue-damaged fruit fly larvae developed allodynia and hyperalgesia (Figure 3A; Babcock et al., 2009). Briefly, the 3rd instar larvae are anesthetized with diethyl ether. Anesthetized larvae are then placed dorsal side up on a microscope slide using two-sided tape and subjected to (mJ/cm^2^) of UV irradiation. After UV exposure, larvae are gently rinsed and placed in a vial containing fly food for 24 h at 25°C. Then larvae are stimulated using a thermal probe. The temperature of thermal probe is set to 41°C to measure for allodynia, and 45°C to detect normal nociception. Withdrawal latency is recorded. After exposure to UV light, injured larvae exhibit heightened behavioral responses to both sub-noxious and noxious stimuli, which suggest that this model serves to effectively investigate both allodynia and hyperalgesia (Babcock et al., 2009; McParland et al., 2021). Using this model, the Hedgehog (Hh), Bone Morphogenetic Protein (BMP), Tumor Necrosis Factor alpha (TNF-α), and Tackykinin (Tk) signaling pathway are found to regulate nociceptive sensitization in response to injury in flies (Babcock et al., 2009; Im et al., 2015; McParland et al., 2021).

A novel adult fly model is developed for a chronic pain analysis process that adult flies show an increase response to a normally threshold temperature (allodynia) after they underwent a leg amputation surgery (Khuong et al., 2019; Massingham et al., 2021). Briefly, the right middle leg of adult fly is amputated at the femur segment using vannas scissors. After amputation, adult flies are fed individually in the vial containing fly food for 7 days. Then, flies are allowed to acclimate to the test chamber on a heating plate. The temperature of the heating plate is raised from 25 to 50°C over 3 min. A video recording camera positioned above the apparatus is used to record observations of flies. Jumping behavior and speed of movement are calculated according to the recorded videos (Figure 3B). This method allows for individualized analysis of allodynia and hyperalgesia.

In general, the current methods used to study the nociception of Drosophila flies are simple and easy to operate, and the equipment is extremely accessible. This makes it easier for researchers to investigate the genetics of acute and chronic pain in human using these tools and assays.

## Development of Anti-Pain Drug Using *Drosophila* Models

*Drosophila melanogaster* are typically used for genetic studies but they also could be employed for drug discovery process (Lee and Min, 2019). The advantages of *D. melanogaster* qualified for drugs screening include the low cost of maintenance, the high reproductive capacity of propagation, and the rapidity of screening studies in the fly compared with traditional rat-based models. It places a high value on investigating new analgesics, especially, with evaluated conserved pain genes, responses and nature of nociception in parallel to human (Manev et al., 2003). Drugs can be delivered to the fruit fly by the following ways such as presented as a vapor (e.g., ethanol and cocaine) (McClung and Hirsh, 1998); either in the food or in the form of a filter paper from sucrose/drug-saturated (Nichols et al., 2002); drug can also be injected or dropped directly onto the exposed nerve cord of flies, of which have been decapitated (Torres and Horowitz, 1998); drugs injected into the abdomen where it quickly diffuses throughout the whole organism can also be available for a valid alternative (Dzitoyeva et al., 2003). In addition, the ability to perform high-throughput screening in flies through random mutation or targeted RNAi-mediated knockdown can further facilitate the identification of new drugs or drug targets (Bell et al., 2009).

Thus, the *Drosophila* fly model for screening putative analgesics appears to be beneficial for the discovery of new drugs. Currently, more and more researchers use fruit flies for pharmacological pain research. Discussions of pain in animals inevitably lead to anthropomorphic references. From a practical standpoint, the animal’s response to noxious stimuli and the ability of drug therapy to attenuate this response are important aspects of pain research. Excitation of gamma-aminobutyric acid B (GABA_*B*_) receptors by injecting agonist 3-aminopropyl-(methyl) phosphinic acid (3-APMPA) significantly prolong latency to heat response in adult flies, and the threshold for heat avoidance enhanced as the injected 3-APMPA concentration increase (Dzitoyeva et al., 2003; Manev and Dimitrijevic, 2004). The peptide Tv1 from *Terebra variegata* has an antinociceptive effect in adult flies, in which injection of Tv1 significantly reduces fly sensitivity to noxious heat (Eriksson et al., 2018). Three analogs of anesthetics (enflurane, isoflurane, and desflurane) can act at a same target as halothane, and decrease the sensitivity to avoid heat in flies that exposed to the heating induced by an intense beam of light (Campbell and Nash, 1994). Paclitaxel as a common chemotherapeutics against cancer can lead to chronic nociception. Consistently, paclitaxel exposure on the fruit fly larval nociception system result in a robust and dose-dependent increase in aversive escape response during a noxious thermal stimulus (Hamoudi et al., 2018). Paclitaxel has also been reported to be toxic in somatic cells, and causes loss of axons in peripheral nerves in *Drosophila* flies (Cunha et al., 2001).

## Concluding Thoughts

As briefly addressed above, there have been several published work in which the fly have been displayed key features that an alternate option biology and physiology, even functional pain genes are well conserved from the fly to humans. The fruit fly applied for pain genomics and pharmacogenomics are devoted in the validation of primary small molecule, the research of the target discovery and the selection of high-throughput screening. However, many factors may participate in pain processes including change of extracellular microenvironment and break of balance in extracellular matrix metabolism, which are never discussed in flies. Pain-like emotions generated by motivational mechanisms are impossible to answer conclusively in flies. As for studies of painkillers in fly, the pharmacological action, the side effects and the best drug-delivery way have not been discussed as to whether they work as well in humans. Although the status of Drosophila as a pain research model is still somewhat different from that of mammals, its potential as a pain research model is being further explored, and its entry into the field of pain research may help reduce the pressure on mammals *in vivo*.

## Author Contributions

JH, BL, SH, YZ, and KL: writing. MX and YL: manuscript editing. All authors contributed to the article and approved the submitted version.

## Conflict of Interest

The authors declare that the research was conducted in the absence of any commercial or financial relationships that could be construed as a potential conflict of interest.

## Publisher’s Note

All claims expressed in this article are solely those of the authors and do not necessarily represent those of their affiliated organizations, or those of the publisher, the editors and the reviewers. Any product that may be evaluated in this article, or claim that may be made by its manufacturer, is not guaranteed or endorsed by the publisher.

## Footnotes

1
http://www.iasp-pain.org
