# Corrigendum: *Drosophila* as a model to study the mechanism of nociception

**DOI:** 10.3389/fphys.2022.956551

**Published:** 2022-07-22

**Authors:** Jianzheng He, Botong Li, Shuzhen Han, Yuan Zhang, Kai Liu, Simeng Yi, Yongqi Liu, Minghui Xiu

**Affiliations:** ^1^ Provincial-Level Key Laboratory for Molecular Medicine of Major Diseases and the Prevention and Treatment With Traditional Chinese Medicine Research in Gansu Colleges and University, Gansu University of Chinese Medicine, Lanzhou, China; ^2^ College of Basic Medicine, Gansu University of Chinese Medicine, Lanzhou, China; ^3^ Key Laboratory for Transfer of Dunhuang Medicine at the Provincial and Ministerial Level, Gansu University of Chinese Medicine, Lanzhou, China; ^4^ College of Integrated Traditional Chinese and Western Medicine, Gansu University of Chinese Medicine, Lanzhou, China; ^5^ State Key Laboratory of Animal Nutrition, College of Animal Science and Technology, China Agricultural University, Beijing, China; ^6^ College of Public Health, Gansu University of Chinese Medicine, Lanzhou, China

**Keywords:** nociception, conserved genetics, nociceptive sensory neurons, behavioral assay, *Drosophila melanogaster*

In the published article, there was an error with the first sentence of **Introduction**, Paragraph Three, being inaccurate. This paragraph previously stated:

“As previous stated, pain as an emotional sensation is unique to humans, and it manifests as the nociceptive pain of *Drosophila* (**Sneddon, 2018**). The nociceptors in the primary afferent nerve fibers are stimulated by thermal, mechanical and chemical stimulation, converted into electrical signals, and then transmitted to the central nervous system such as the spinal cord, and finally felt the pain (**Julius, 2013**; **Bourne et al., 2014**; **Dai, 2016**; **Sneddon, 2018**; **St, 2018**). These nerve fibers quickly transmit the perceived harmful information to the central nervous system through action potentials. In this process, ion channels play a vital role. These ion channels are specifically expressed in the above-mentioned nerve fibers (**Julius, 2013**; **Dai, 2016**; **Yam et al., 2018**). TRP, Piezo and other ion channels have been identified as key pain receptors (**Hwang and Oh, 2007**; **Volkers et al., 2015**). Among these channels, TRPV1, TRPA1, Piezo1 and Piezo2 are expressed in nociceptors (**Liedtke, 2007**; **Flood et al., 2013**; **Volkers et al., 2015**; **Himmel and Cox, 2017**; **Boonen et al., 2021**). They serve as detectors and sensors for cold, heat, chemical and mechanical stimuli in nociceptors. These conserved genes in *Drosophila* well prove the potential of flies as a nociceptive model animal. The purpose of this review is to present the aggregate findings of the pain-related genes in order to discuss the possibilities for *Drosophila* as model animal in nociception research, and provide a comprehensive evaluation for future human nociception studies.”

The corrected paragraph appears below:

“As previously mentioned, there are conserved physiological mechanisms underlying the nociceptive system between human and flies (**Sneddon, 2018**). The nociceptors in the primary afferent nerve fibers are stimulated by thermal, mechanical and chemical stimulation, converted into electrical signals, and then transmitted to the central nervous system such as the spinal cord, and finally felt the pain (**Julius, 2013**; **Bourne et al., 2014**; **Dai, 2016**; **Sneddon, 2018**; **St, 2018**). These nerve fibers quickly transmit the perceived harmful information to the central nervous system through action potentials. In this process, ion channels play a vital role. These ion channels are specifically expressed in the above-mentioned nerve fibers (**Julius, 2013**; **Dai, 2016**; **Yam et al., 2018**). TRP, Piezo and other ion channels have been identified as key pain receptors (**Hwang and Oh, 2007**; **Volkers et al., 2015**). Among these channels, TRPV1, TRPA1, Piezo1 and Piezo2 are expressed in nociceptors (**Liedtke, 2007**; **Flood et al., 2013**; **Volkers et al., 2015**; **Himmel and Cox, 2017**; **Boonen et al., 2021**). They serve as detectors and sensors for cold, heat, chemical and mechanical stimuli in nociceptors. These conserved genes in *Drosophila* well prove the potential of flies as a nociceptive model animal. The purpose of this review is to present the aggregate findings of the pain-related genes in order to discuss the possibilities for *Drosophila* as model animal in nociception research, and provide a comprehensive evaluation for future human nociception studies.”

The authors apologize for this error and state that this does not change the scientific conclusions of the article in any way. The original article has been updated.

